# Cocaine treatment admissions at three sentinel sites in South Africa (1997–2006): findings and implications for policy, practice and research

**DOI:** 10.1186/1747-597X-2-37

**Published:** 2007-12-28

**Authors:** Charles DH Parry, Andreas Plüddemann, Bronwyn J Myers

**Affiliations:** 1 Alcohol and Drug Abuse Research Unit, Medical Research Council, Cape Town, South Africa; 2 Department of Psychiatry, Stellenbosch University Medical School, Cape Town, 7505, South Africa

## Abstract

**Background:**

Accurate prevalence data on cocaine use, that points to where problems exist and the extent of these problems, is necessary to guide the formulation of effective substance abuse policy and practice. The purpose of this study was to provide surveillance information about the nature and extent of problematic cocaine use in South Africa.

**Methods:**

Data were collected between January 1997 and December 2006 on admissions for drug abuse treatment through a regular monitoring system involving 56 drug treatment centres and programmes in Cape Town, Gauteng Province (Johannesburg and Pretoria) and the Eastern Cape every six months as part of the South African Community Epidemiology Network on Drug Use (SACENDU). A one-page form was completed by treatment centre personnel to obtain demographic data, the patients' primary and secondary substances of abuse, the mode, frequency and age of first use of substance, and information on prior treatment.

**Results:**

Treatment indicators point to a significant increase in cocaine related admissions over time in all sites, but with substantial inter-site variation, particularly in recent years. The data indicate high levels of crack cocaine use and high levels of daily usage among patients, most of whom were first time admissions. Patients with cocaine related problems continue to be predominantly male, with a mean age of around 30 years. Substantial changes in the racial profile of patients have occurred over time. Poly drug use is high with cocaine often used with alcohol, cannabis and other drugs.

**Conclusion:**

These trends point to the possibility of cocaine use becoming a serious health and social issue in South Africa and demonstrate the utility of continued monitoring of cocaine treatment admissions in the future. They also highlight the need to address cocaine use in national and provincial policy planning and intervention efforts. In terms of treatment, the findings highlight the need to ensure that treatment practitioners are adequately trained to address stimulant problems, poly drug use, and HIV and other risk behaviour related to crack cocaine use. Possible gaps in access to treatment by certain sectors of the population should be addessed as a matter of urgency.

## Background

According to the European Monitoring Centre for Drugs and Drug Addiction (EMCDDA), cocaine use is currently at historically high levels in Europe, with new treatment admissions for cocaine more than doubling between 1999 and 2005 [1]. In Africa, particularly in countries in the west and south-east, cocaine use has also increased [2]. South Africa, one of the largest countries in Africa with a population of 45 million [3] and a driving force behind The New Partnership for Africa's Development (NEPAD) has seen a rapid increase in cocaine, heroin and amphetamine type stimulant trafficking and use since independence from white minority rule ('Apartheid') in 1994. This increase has been ascribed to the opening up of the country's borders, a decrease in very restrictive internal state controls, high levels of unemployment, and an increasing use of this country as a route for transshipment of cocaine from South America to Europe and occasionally North America [4].

In South Africa there have been few national surveys to assess the extent of cocaine use since 1994. While the most recent national survey conducted among persons 15 years and older in 2005 found that 0.3% of participants reported lifetime use of cocaine [5], the Youth Risk Behaviour Survey conducted in 2002 among students in grades 8–11 reported a lifetime prevalence of 6.4% [6]. The use of cocaine potentially has important implications for public health in South Africa with its already high levels of violent crime and HIV infection [4,7,8]. Accurate prevalence data on cocaine use, that points to where problems exist and the extent of such problems, is necessary to guide the formation of effective drug policies and practices. While national surveys have a number of advantages, on their own they have significant limitations. These include a limited ability to estimate the prevalence of less commonly used drugs like cocaine, especially if sample sizes are small. In contrast, datasets on treatment admissions are useful for indirectly assessing trends in problematic drug use, and have been used for this purpose among others in the European Union and other parts of the world [9-11].

Given reports of the increasing transshipment of cocaine via Africa to Europe and EMCDDA reports of increasing numbers of individuals presenting for treatment with cocaine related problems, our aim was to determine if treatment admissions for cocaine related problems were also increasing in South Africa, a country with strong historical and economic ties to countries in the European Union. Specifically our purpose was to describe trends in treatment admissions for cocaine related problems in three sentinel sites in South Africa for the period January 1997 to December 2006 and compare South African findings with the most recent data on cocaine related treatment admissions from the EMCDDA.

## Methods

Established in 1996, the South African Community Epidemiology Network on Drug Use (SACENDU) is a network of researchers, practitioners and policy makers from eight sentinel sites in South Africa who meet bi-annually to provide community-level public health surveillance information about alcohol and other drug (AOD) trends. These sites consist of the two port cities of Cape Town and Durban and six provinces: Gauteng, which includes the cities of Johannesburg and Pretoria (added in 1998); Mpumalanga, in the northeast of the country (added in 1999); the Eastern Cape, comprising the urban centres of Port Elizabeth, East London, Umtata, and surrounding areas (added in 2004); and the North West, the Northern Cape and the Free State provinces (all added in 2006). Together they comprise about 75% of the population of the country.

The focus of this paper is on data related to cocaine related problems obtained from specialist AOD treatment centres in Cape Town, Gauteng and the Eastern Cape. These sites were chosen as they collectively have the largest number of treatment centres (56 of the 73 centres participating across the eight site monitoring system) and account for roughly 40% of the total population of the country [3]. Currently specialist treatment centres are the main source of data on cocaine use for SACENDU. Data are currently collected from all state-funded and most private institutions, including more than 80% of treatment centres in these sites (28 in Cape Town, 19 in Gauteng, and 9 in the Eastern Cape) [12,13]. In order to be admitted to alcohol and other drug treatment centres, patients are generally required to meet the *Diagnostic and Statistical Manual *version IV (DSM-IV) criteria for substance abuse or substance dependence.

For the purpose of surveillance, a standardised one-page form is completed on each person treated by a given centre during a particular six-month period. The form elicits responses about the source of referral for treatment, demographic information, the type of treatment received (inpatient and/or outpatient), the primary, secondary, tertiary and fourth substance of abuse, the mode(s) of use, frequency of use, age of first use and whether the person had received treatment prior to the current episode. The items included in the form were drawn from items used by the Pompidou Group in Europe in the mid 1990s and are similar to items currently included in the EMCDDA's Treatment Demand Indicator Protocol (TDI) and the US Treatment Episode Data Set (TEDS) [10,11]. Treatment centres receive ongoing training in data collection procedures. Typically the form is filled in by the case manager a few days after the patient has been admitted to the centre. To ensure data quality, completed forms are checked for missing information and possible miscodes. Ethical approval for the SACENDU project was granted by the Ethics Committee of the Medical Research Council of South Africa.

In terms of data analysis, descriptive statistics are reported together with Cochrane-Armitage tests to assess whether particular trends are statistically significant.

## Results

Data on the proportion of patients having cocaine as a primary or overall (primary to fourth) drug of abuse across the three sites is given in Table 1, together with the Cochrane-Armitage trend test results for each distribution. The cumulative total number of patients seen at these AOD treatment centres for any substance of abuse, including alcohol, over the 20 data collection periods, was 38,686 in Cape Town (between January 1997 and December 2006), 49,171 in Gauteng (between January 1998 and December 2006) and 3,939 in the Eastern Cape (between January 2004 and December 2006). The Cochrane-Armitage trend tests revealed that the proportion of patients reporting cocaine as their primary drug of abuse relative to other substances, increased significantly in all sites over time (Table 1).

**Table 1:** Percentage of patients from specialist treatment centres in Cape Town, Gauteng and Eastern Cape with cocaine (HCL/crack) as a primary or overall^1 ^drug of abuse, ratio of crack users to total cocaine users as a %, and % of new admissions among patients with cocaine as a primary drug of abuse

	Cape Town						Gauteng						Eastern Cape					
	Users in treatment for cocaine or crack (primary)		% of crack to total cocaine	% of users reporting cocaine overall^1^		% new adm.^3,4^	Users in treatment for cocaine or crack (primary)		% of crack to total cocaine	% of users reporting cocaine overall^1^		% new adm.^3,4^	Users in treatment for cocaine or crack (primary)		% of crack to total cocaine	% of users reporting cocaine overall^1^		% new adm.^3,4^

	n	%		n	%		n	%		n	%		n	%		n	%	

1997a	71	3.4	85.9	91	4.3	-	-	-	-	-	-	-	-	-	-	-	-	-
1997b	78	3.6	64.1	117	5.4	-	-	-	-	-	-	-	-	-	-	-	-	-
1998a	131	5.7	78.6	265	11.5	-	157	8.0	52.5	271	12.8	-	-	-	-	-	-	-
1998b	99	7.3	65.7	198	14.5	-	204	8.6	79.6	374	15.8	-	-	-	-	-	-	-
1999a	126	8.3	72.2	168	11.0	-	283	10.3	78.6	453	16.5	-	-	-	-	-	-	-
1999b	137	8.8	70.1	299	19.3	61.3	273	10.5	78.1	491	18.8	-	-	-	-	-	-	-
2000a	132	7.8	65.9	284	16.8	61.2	267	10.6	77.4	430	17.1	-	-	-	-	-	-	-
2000b	121	7.1	67.8	252	14.9	59.5	198	7.5	80.0	383	14.3	59.6	-	-	-	-	-	-
2001a	144	9.2	68.1	335	21.3	54.8	210	7.4	77.0	466	16.4	-	-	-	-	-	-	-
2001b	88	5.6	73.9	231	14.8	57.5	155	5.8	74.1	386	14.4	52.9	-	-	-	-	-	-
2002a	106	6.6	52.8	211	13.1	63.2	183	5.2	84.6	429	14.6	59.6	-	-	-	-	-	-
2002b	104	6.7	58.7	247	15.9	67.0	146	5.6	67.9	355	13.7	54.8	-	-	-	-	-	-
2003a	136	7.9	72.1	312	18.1	55.9	155	5.9	66.1	381	14.6	51.0	-	-	-	-	-	-
2003b	139	8.4	66.9	358	21.6	61.6	182	6.8	70.6	394	14.5	56.6	-	-	-	-	-	-
2004a	219	9.7	65.8	491	21.8	59.4	256	9.1	72.3	507	18.0	69.5	33	5.1	48.5	76	11.6	69.7
2004b	210	9.1	67.1	463	20.1	63.8	263	9.9	68.4	510	19.2	63.1	42	7.0	31.0	82	13.7	47.6
2005a	205	8.3	62.9	475	19.2	69.3	271	9.0	69.0	577	19.0	63.1	73	10.9	65.8	125	18.6	62.5
2005b	163	7.6	69.3^2^	386	18.1	71.1	287	10.1	69.3	574	20.2	71.4	53	9.1	75.5^2^	88	15.0	71.7
2006a	159	6.0	65.4^2^	415	15.6	59.1	345	11.1	71.3	667	21.4	64.1	145	18.4	78.2^2^	218	27.7	63.7
2006b	134	4.8	62.5^2^	346	12.4	57.5	353	10.7	67.1	778	23.6	68.3	124	19.2	83.9^2^	187	29.0	65.3
Trend test	Z = 4.461			Z = 15.982			Z = 3.775			Z = 11.964			Z = 9.941			Z = 2.578		
Sigif.	p < 0.0001			p < 0.0001			p < 0.0001			p < 0.0001			p < 0.0001			p < 0.005		

- data not recorded at that site; ^1 ^includes cocaine or crack as 1^st^, 2^nd^, 3^rd ^or 4^th ^substance of abuse; ^2 ^distinction between cocaine and crack no longer recorded, have used mode as a proxy with "smoking" taken as indicative of crack cocaine use; ^3 ^primary substance of abuse; ^4 ^% new admissions among cocaine/crack patients

**Figure 1:**
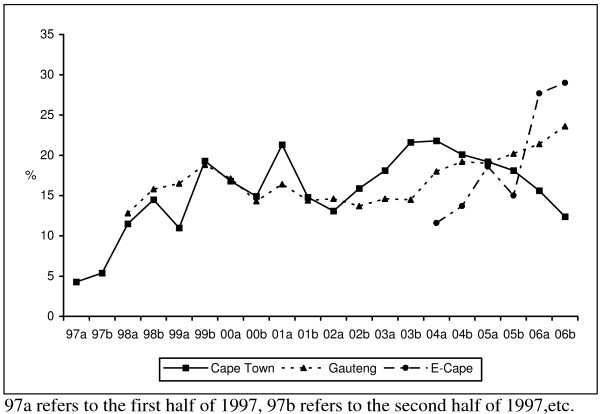
Percentage of patients from specialist treatment centres in Cape Town, Gauteng and the Eastern Cape reporting that cocaine was a primary, secondary, tertiary or fourth drug of abuse, January 1997 to December 2006 (by 6-month period).

The trend tests for the proportion of users in each of the three sites having cocaine as an overall drug of abuse were also statistically significant. The trend tests, however, mask the actual distribution of patients that had cocaine as an overall substance of abuse (Figure 1). For Cape Town, the graph shows an increase in treatment admissions for cocaine related problems from 1997 until the second half of 1999, then a decline until the first half of 2002 (with an isolated increase in the first half of 2001), then an increase until the first half of 2004, after which it gradually starts to decline. The graph for Gauteng also shows an increase till the second half of 1999 and then a decline until the second half of 2003 with steady increases thereafter. With the exception of the second half of 2005, the graph for the Eastern Cape has shown a steady increase in cocaine related treatment admissions since the start of data collection.

Over time the percentage of crack cocaine use relative to cocaine hydrochloride (HCL) use has fluctuated between just over 50% to around 85% in Gauteng and Cape Town and between 31% and 66% in the Eastern Cape. The ratio of crack cocaine to cocaine HCL is currently about two to one in Cape Town and Gauteng, and more than four to one in the Eastern Cape (Table 1).

In all sites the percentage of first time admissions among patients having cocaine as a primary drug of abuse has fluctuated between about 50% and 70% (Table 1). There has been no discernable increase or decrease in this percentage over time. Among patients who present for the treatment of cocaine related problems, the main mode of cocaine HCL administration is through snorting, and crack cocaine is smoked. Across all sites less than one percent of patients reported injecting cocaine. This has not changed over time. In terms of the frequency of cocaine use, 65% of patients in treatment in Cape Town with cocaine as a primary drug of abuse in the second half of 2006 reported daily use, compared to 45% in the Eastern Cape. A quarter of these patients in Cape Town reported using cocaine between two and six times per week compared to 41% in the Eastern Cape. In the second half of 2006 the mean length of time between the age of first treatment admission and age of first use of cocaine was 3.4 years (SD = 4.68) in the Eastern Cape and 6.1 years (SD = 5.25) in Cape Town. No data were available on this for Gauteng.

The mean age at which patients with cocaine related problems present for treatment has remained between 29 and 32 years in Cape Town (overall mean = 29.9, SD = 0.86), 28 and 30 years in Gauteng (overall mean = 28.7, SD = 0.75), and 27 and 29 years in the Eastern Cape (overall mean = 28.1, SD = 0.78). Table 2 shows that from 1997 to 2006 the percentage of persons seeking treatment for cocaine related problems who were female has fluctuated between 10% and 31% in Cape Town (overall mean = 21.8, SD = 4.55), between 14% and 28% in Gauteng (overall mean = 20.6, SD = 3.82), and between 14% and 20% in the Eastern Cape (overall mean = 17.0, SD = 2.12).

**Table 2:** Gender and racial profile of patients with cocaine (HCL/crack) as their primary drug of abuse: 1997–2006

	Cape Town					Gauteng					Eastern Cape				
Period	Fem.	Black Afr.	Coloured	Asian	White	Fem.	Black Afr.	Coloured	Asian	White	Fem.	Black Afr.	Coloured	Asian	White

1997a	19.7	0.0	3.6	7.1	89.3	-	-	-	-	-	-	-	-	-	-
1997b	31.2	2.3	25.6	2.3	69.8	-	-	-	-	-	-	-	-	-	-
1998a	20.6	1.4	23.9	2.8	71.8	17.8	-	-	-	-	-	-	-	-	-
1998b	20.2	5.1	17.0	1.7	76.3	17.2	-	-	-	-	-	-	-	-	-
1999a	22.0	1.3	32.9	2.5	63.3	24.0	-	-	-	-	-	-	-	-	-
1999b	19.1	1.3	38.2	13.2	47.4	23.4	4.8	8.8	12.8	73.6	-	-	-	-	-
2000a	9.8	0.9	51.9	4.6	42.6	17.2	3.8	9.3	12.8	73.7	-	-	-	-	-
2000b	19.2	0.0	38.8	2.0	59.2	19.7	8.0	7.6	13.1	69.2	-	-	-	-	-
2001a	25.0	2.6	29.3	4.3	63.8	19.5	6.2	8.6	8.6	76.6	-	-	-	-	-
2001b	19.3	1.1	43.7	5.7	49.4	23.2	7.7	12.9	9.0	70.3	-	-	-	-	-
2002a	20.8	5.8	29.1	5.8	59.2	27.9	4.9	7.7	9.8	77.6	-	-	-	-	-
2002b	28.8	1.0	32.4	5.9	60.8	17.1	10.3	8.2	10.3	71.2	-	-	-	-	-
2003a	18.3	1.5	37.0	11.9	49.6	27.1	7.7	7.1	8.4	76.8	-	-	-	-	-
2003b	19.6	6.6	45.6	2.9	44.9	22.0	10.4	8.8	10.4	70.4	-	-	-	-	-
2004a	21.0	3.7	42.8	4.2	49.3	18.0	9.4	15.6	7.4	67.6	18.2	3.0	15.2	12.1	69.7
2004b	22.4	2.4	40.3	3.9	53.4	22.4	9.2	14.1	5.3	71.1	14.3	4.8	28.6	2.4	64.3
2005a	24.6	2.0	46.6	4.9	45.6	21.0	8.5	19.6	6.6	65.3	15.1	5.5	32.9	5.5	56.2
2005b	27.6	3.7	47.2	1.2	47.9	23.3	11.5	20.6	3.5	64.4	17.0	1.9	34.0	3.8	60.4
2006a	25.2	4.5	38.5	3.8	53.2	13.6	12.8	16.2	7.8	63.2	20.2	2.4	34.7	6.5	56.5
2006b	21.1	3.8	31.1	6.1	59.1	17.0	12.5	18.7	9.1	59.8	16.9	3.2	43.5	6.5	46.8

In Cape Town the proportion of black African patients having cocaine as a primary drug of abuse has been consistently low (under 7%) whereas there has been an increase in the proportion of cocaine patients who are "Coloured" over time (Z = 4.003, *p *< 0.0001) (Table 2). The latter has resulted from a decline in the proportion of cocaine patients who are white. The terms "white", "black", and "Coloured", originate from the Apartheid era. They refer to demographic markers and do not signify inherent characteristics. They refer to people of European, African and mixed (African, European and/or Asian) ancestry, respectively. Their continued use in South Africa is important for monitoring improvements in health and socio-economic disparities, identifying vulnerable sections of the population, and planning effective interventions.

In Gauteng between the second half of 1999 and the second half of 2006 roughly 60% or more patients having cocaine as a primary drug of abuse were white (Table 2). The Cochrane-Armitage trend test revealed that the proportion of patients treated for cocaine related problem who were black African in Gauteng increased significantly from 4.8% in the second half of 1999 to 12.5% in the second half of 2006 (*Z *= 5.364, *p *< 0.0001). Similarly there was a significant increase in the proportion of cocaine patients who were Coloured, from 8.8% to 18.7% (*Z *= 6.985, *p *< 0.0001). In the Eastern Cape there was also a significant increase in the proportion of Coloured cocaine patients, from 15.2% in the first half of 2004 to 43.5% in the second half of 2006 (Z = 5.218, *p *< 0.0001). Likewise the proportion of cocaine patients who were Coloured in Cape Town increased significantly over the 20 reporting periods from 3.6% to 31.1% (Z = 4.003, *p *< 0.0001).

Poly drug use is common. Table 3 shows for the second half of 2006 for each site separately both the proportion of other drugs that are secondary when cocaine is primary as well as the primary drugs of abuse when cocaine is secondary. When cocaine is the primary drug of choice, it is often used in conjunction with alcohol and with cannabis. In the Cape Town and the Eastern Cape the main "sedative" drug used in combination with cocaine (when primary) appears to be methaqualone (a sedative-hypnotic known locally as Mandrax), whereas in Gauteng it is heroin. In Cape Town the main primary drug that was reported when cocaine was secondary was methamphetamine (another stimulant), in Gauteng it was heroin (a "downer") and in the Eastern Cape it was methaqualone (a sedative). A number of secondary cocaine users in all sites used heroin as primary drugs of abuse. In Cape Town methamphetamine also featured prominently.

**Table 3:** Proportion of selected other drugs abused with cocaine as primary and secondary drug of abuse (2006b)

	Cape Town		Gauteng		Eastern Cape	
	Cocaine as primary drug: Other drugs	Drugs that are primary when cocaine is secondary	Cocaine as primary drug: Other drugs	Drugs that are primary when cocaine is secondary	Cocaine as primary drug: Other drugs	Drugs that are primary when cocaine is secondary

None	30.6	N/A	36.5	N/A	41.1	N/A
Alcohol	37.3	26.8	29.2	25.4	25.8	18.6
Cannabis	26.9	13.2	23.2	20.4	29.0	32.6
Methaqualone	12.7	3.4	3.7	2.6	18.5	11.6
Heroin	6.0	18.5	10.5	35.0	5.7	20.9
Ecstasy	6.0	1.5	6.2	1.5	7.3	7.0
Methamphetamine	11.9	33.7	0.6	0.5	1.6	7.0
Methcathinone	0.8	1.0	4.0	13.1	0	1.6
OTC-Prescription medicines	6.7	1.5	4.8	1.5	1.6	2.3

## Discussion

Findings from SACENDU indicate that over time cocaine use has placed an increased burden on AOD treatment services in several provinces in South Africa, though some inter-site variation is occurring. This increase has not been constant and at times cocaine related treatment admissions have even shown a decline. The drop off in cocaine related treatment admissions in Cape Town and Gauteng in the second half of 1999 and the subsequent increase might be linked to changes in global cocaine production over that time period [14]. In all sites reported on in this paper, treatment admissions related to cocaine use have increased over time, but the current rate of increase appears to be much higher in the Eastern Cape than in Gauteng and Cape Town. There have been no systematic changes in the provision of treatment services or admissions policies that could explain these findings. Discussions with community sources and the police indicate that the increase in cocaine related admissions in the Eastern Cape is mainly due to the recent emergence of the cocaine trade in Port Elizabeth and East London and the very aggressive marketing practices accompanying it. If only national data had been reported on, local variations such as those recently experienced in the Eastern Cape and in Cape Town would have disappeared.

The overall increase in cocaine related treatment admissions over time has been accompanied by a reduction in treatment admissions for other drugs of abuse with depressant qualities, specifically alcohol and methaqualone [15]. It has also been accompanied by an increase in admissions for a broad range of other stimulants including methamphetamine (in Cape Town) and methcathinone (in Gauteng) [15]. A similar rise in treatment admissions related to cocaine use was indicated in the 2007 Annual Report of the EMDCCA on trend data for 1999 through 2005, increasing from 11% to 24% of new drug clients during this period [18]. In contrast, in the USA there appears to have been a steady fall off in cocaine related treatment admissions since the mid 1990s, accompanied by a rise in problems related to other stimulants and opiates [16]. The recent decline in cocaine related treatment admissions in Cape Town is in all likelihood also due to the increase in treatment admissions related to another stimulant, methamphetamine [17]. In both South Africa and the European Union, the majority of patients coming to treatment with cocaine as a primary drug of abuse are new admissions. In the absence of other systemic changes, this may reflect an increasing incidence of cocaine related problems.

In order to form effective prevention and treatment programmes the demographic profile of cocaine users needs to be identified. Persons in treatment for cocaine for the first time across the three South African sites are aged between 27 and 32 years and are generally older than individuals with problems related to other drugs [9]. They generally started using cocaine three to five years prior to treatment. Similar findings were reported by the EMCDDA, where in 2004 the mean age for new patients entering outpatient treatment for cocaine was 31 years for males and 28 years for females. They were also reported as being typically older than other drug consumers, apart from users of hypnotics and sedatives, who are the oldest even if figures are low [18]. In the USA the average age of primary cocaine admissions was 38 years for smoked cocaine and 34 years for non-smoked cocaine [16]. The older age of cocaine patients may have implications for issues that will need to be addressed during treatment, such as a greater focus on the effect of their drug use on partners, children and work than perhaps would be needed for patients in their late teens and early twenties.

Substantially more males than females accessed treatment for cocaine problems. The male to female ratio for patients entering outpatient treatment for cocaine as a primary drug of abuse in 15 European countries in 2004 was 5.7:1 [18] which is fairly similar to what was found in this study.

Rather than reflecting lower levels of cocaine use among women, this may partly reflect gender differences in access to treatment [19]. There seems to be a greater stigma associated with drug dependence in females, and the abuse of illicit drugs tends to remain hidden. In addition, women in South Africa often do not have an independent income to pay for treatment. Nevertheless, about 10%–30% of persons treated for cocaine abuse are female.

Furthermore, the data suggest an increase in cocaine related treatment admissions by Coloureds in all three sites and by black Africans in Gauteng. In the 1990s drug markets in South Africa were clearly segmented along racial lines, with drugs like cocaine being marketed to whites who tended to be more affluent [20]. Since 2000, these markets have become less segmented, as reflected in the aforementioned demographic shifts. The changes over time cannot be explained in terms of systematic changes in service delivery that might have increased access by particular population groups. The likely reason that black Africans are not showing up in greater numbers at drug treatment centres in the Eastern Cape is probably due to the substantially higher levels of poverty [3] which has meant that black Africans in this province tend to abuse alcohol and cannabis rather than more expensive substances, as well as the limited number of treatment slots in the affordable, non-profit treatment sector. Lower utilisation of drug treatment services for cocaine and other drug problems by black Africans in Cape Town and Gauteng could also be due to the fact that the majority of black Africans still reside in suburbs far from drug treatment and other services [21].

In contrast to Europe where more than 80% of new outpatients with cocaine problems report using cocaine hydrochloride [18], in South Africa the most prominent mode of cocaine consumption reported by persons coming to drug treatment is crack cocaine (65% to 84% in the three sites currently). Treatment data from the USA also indicates high levels of crack cocaine usage (72% in 2005). There are indications that the use of cocaine in this form increases sexual risk behaviour and is related to levels of violence [22,23]; although these associations could also be partially explained by contextual factors such as crowding. The growing use of crack cocaine is of great concern to a country like South Africa where sexual risk behaviour and interpersonal violence together contribute 40% of the total burden of death and disability in the country [24]. With high levels of patients reporting daily use of cocaine (e.g. 65% of patients in Cape Town) this is an added concern. With regard to mode of use, roughly 70% of patients in treatment reported smoking their cocaine with the remainder mostly snorting/sniffing. Smoking is an extremely potent and direct form of administration. In contrast, EMCDDA treatment data for 2005 reported that 53% of patients in treatment reported snorting or sniffing the substance, 34% smoking it and 5% injecting [18]. Fortunately less than one percent of patients in the South African study reported injecting cocaine, thus decreasing their risk of contracting injection-related HIV or Hepatitis B/C.

In responding to the threat of cocaine use in South Africa it is important to remember that poly-drug use is high. In roughly two-thirds of cases where cocaine was the primary drug of abuse other drugs were reported as secondary, typically alcohol and cannabis. Similar secondary drugs were reported by the EMCDDA with the addition of heroin [18]. Conversely, in Gauteng and the Eastern Cape a high proportion of patients having heroin as a primary drug of abuse had cocaine as a secondary drug. Treatment planning needs to take into account such poly-drug use. According to the most recent European data a diversification of cocaine users in treatment can be identified. Three main groups are seen, (i) users of cocaine powder often combined with alcohol and/or cannabis, (ii) users of crack cocaine with other drugs and/or heroin, and (iii) polydrug users, consuming cocaine and heroin [18].

While political attention in South Africa has been given increasingly to problems related to methamphetamine and heroin use, very little attention has been directed to cocaine abuse despite the fact that roughly one in five patients coming to drug treatment currently has cocaine as a primary or secondary drug of abuse. One of the policy implications of this research is that substance abuse practitioners need to be trained in and provided with specific treatment protocols for addressing cocaine and other stimulant related problems. Such protocols and training are lacking in South Africa. The increasing levels of cocaine related treatment admissions in Gauteng and the Eastern Cape also highlight the importance of addressing gaps in supply reduction and ensuring that universal and selected prevention programmes take cognisance of particular issues related to cocaine use. Prevention efforts need to focus on persons who have not used any drugs as well as persons in their early twenties who might be using other drugs. Given the ongoing rise in use of cocaine among Coloured and black African populations, prevention efforts need to be sensitive to pressures to use cocaine among these population groups and in communities where they tend to reside.

The main limitation of this study is that treatment data are affected by the lack of available treatment options for drug abuse in South Africa, particularly for the most disadvantaged sectors of society [21]. This limitation highlights the need for further community-based studies such as school surveys, key informant and user interviews to assess the use of the drug in populations that might not have access to specialist substance abuse treatment or who might be accessing other services such as private mental health professionals and other kinds of support services. While we controlled for double counting of patents within a treatment centre we were not able to control for double counting across centres. This may have slightly inflated the number of patients receiving treatment and may have biased the data towards those patients who seek treatment across more than one institution. Another limitation of the study is that not all the patients would necessarily have met the criteria for cocaine dependence. Further research is therefore required that would investigate the severity of the problems experienced by patients seeking treatment for cocaine problems. A further limitation of this study comes from the fact that data were only reported on three sites. It is possible that other patterns of cocaine use exist in other areas. With the expansion of SACENDU to all provinces in 2007/8, this shortcoming will be addressed.

## Conclusion

In South Africa, as in Europe, there has been a substantial increase in cocaine related treatment admissions over time. In the second half of the 1990s cocaine users entering treatment tended to be white males who preferred to smoke the substance. Treatment admissions were traditionally higher in large urban centres like Cape Town and Pretoria/Johannesburg (Gauteng province). In recent years, a partial shift in this profile has occurred, with use increasing in less economically strong areas like the Eastern Cape, and among black African and especially Coloured users. These emerging trends point to the possibility of cocaine use becoming a serious health and social issue in South Africa and demonstrate the need for continued monitoring of cocaine treatment admissions in the future (especially given the dearth of any other ongoing monitoring systems) and demonstrate the need to address cocaine use in national and provincial policy planning and intervention efforts.

## Competing interests

The author(s) declare that they have no competing interests.

## Authors' contributions

CP and AP run the South African Community Epidemiology Network on Drug Use (SACENDU), CP since 1996 and AP since 2000. BM has been involved in analysis and write up of SACENDU data since 2001. All three authors were involved in the analysis of the data on which this paper was written, revised drafts of the manuscript and approved the final manuscript.
